# A chaotic view of behavior change: a quantum leap for health promotion

**DOI:** 10.1186/1479-5868-3-25

**Published:** 2006-09-12

**Authors:** Ken Resnicow, Roger Vaughan

**Affiliations:** 1 University of Michigan, Department of Health Education and Health Behavior, School of Public Health, Ann Arbor, MI, USA; 2 Department of Biostatistics, Columbia University, 722 West 168th Street, 6th Floor, New York, NY 10032, USA

## Abstract

**Background:**

The study of health behavior change, including nutrition and physical activity behaviors, has been rooted in a cognitive-rational paradigm. Change is conceptualized as a linear, deterministic process where individuals weigh pros and cons, and at the point at which the benefits outweigh the cost change occurs. Consistent with this paradigm, the associated statistical models have almost exclusively assumed a linear relationship between psychosocial predictors and behavior. Such a perspective however, fails to account for non-linear, quantum influences on human thought and action. Consider why after years of false starts and failed attempts, a person succeeds at increasing their physical activity, eating healthier or losing weight. Or, why after years of success a person relapses. This paper discusses a competing view of health behavior change that was presented at the 2006 annual ISBNPA meeting in Boston.

**Discussion:**

Rather than viewing behavior change from a linear perspective it can be viewed as a quantum event that can be understood through the lens of Chaos Theory and Complex Dynamic Systems. Key principles of Chaos Theory and Complex Dynamic Systems relevant to understanding health behavior change include: 1) Chaotic systems can be mathematically modeled but are nearly impossible to predict; 2) Chaotic systems are sensitive to initial conditions; 3) Complex Systems involve multiple component parts that interact in a nonlinear fashion; and 4) The results of Complex Systems are often greater than the sum of their parts. Accordingly, small changes in knowledge, attitude, efficacy, etc may dramatically alter motivation and behavioral outcomes. And the interaction of such variables can yield almost infinite potential patterns of motivation and behavior change. In the linear paradigm unaccounted for variance is generally relegated to the catch all "error" term, when in fact such "error" may represent the chaotic component of the process. The linear and chaotic paradigms are however, not mutually exclusive, as behavior change may include both chaotic and cognitive processes. Studies of addiction suggest that many decisions to change are quantum rather than planned events; motivation arrives as opposed to being planned. Moreover, changes made through quantum processes appear more enduring than those that involve more rational, planned processes. How such processes may apply to nutrition and physical activity behavior and related interventions merits examination.

## Background

"What we call **chaos **is just patterns we haven't recognized. What we call random is just patterns we can't decipher. What we can't understand we call nonsense. What we can't read we call gibberish"

Chuck Palahniuk

The study of health behavior change, including nutrition and physical activity behaviors, has historically been rooted in a cognitive-rational paradigm. Extant models, such as Social Cognitive Theory, the Health Belief Model, the Theory of Planned Behavior, the Transtheoretical Model and others, have generally viewed change as an interaction of cognitive factors such as knowledge, attitude, belief, efficacy and intention [1,2]. Change is conceptualized as a linear, deterministic process where individuals weigh the pros and cons, and at the point at which the benefits outweigh the cost, "decisional balance" tips them toward change. An implicit assumption within this perspective is that change is a gradual process under conscious control. Consistent with this framework, the associated statistical models have almost exclusively assumed a linear relationship between psychosocial predictors and behavior (change); i.e., greater increases in knowledge, attitudes and intentions will lead to greater change in behavior.

However, the theoretical and statistical assumptions underlying this linear paradigm may be seriously flawed. In particular, such a perspective fails to account for non-linear, quantum influences on human thought and action. The limitations of a rational-linear conceptualization of behavior change may in part (in addition to measurement error) explain the modest proportion of behavioral variance accounted for by such models; which typically has been in the range of around 10%–20% and rarely higher than 50% [3-11]. The fact that the majority of studies have employed cross-sectional designs and relied on self-report to measure behavior further suggests that the true variance accounted for by linear models may be even lower [12]. Below we provide an alternative model of health behavior change based on non-linear dynamics.

## Discussion

An alternative view is that decisions to initiate (and possibly maintain) behavior change are quantum rather than linear events [13]. Such quantum leaps result from a surge of motivation or inspiration that is greater than the sum of its cognitive parts. It is not so much a planned decision, but something that arrives beyond cognition. The more dramatic form of quantum change is described by Miller [14]:

"Buried in the statement "I just decided", however can be another kind of experience that has been confused with ordinary decision making. It is the insightful type of quantum change. When people talk about such experiences in shorthand, they may say "it just happened" or "I just decided". Inquire a little more closely, however, and it becomes apparent that the process is somewhat more complex." (page 37)

Miller delineates two types of quantum change, sudden insights and mystical epiphanies. Both kinds leave an indelible impact and often lead to lasting and pervasive change. Both usually involve a significant alteration in how the person perceives him/her self, others and the world. Although the cases described in Miller's book tend to involve an overwhelming transformation, less dramatic, less mystical "mini-epiphanies" may contribute to many behavior change decisions. From this perspective, behavior change can be understood through the lens of Chaos Theory and Complex Dynamic Systems. Four key principles from these theories relevant to understanding health behavior change are:

1) Chaotic systems can be mathematically modeled, usually in non-linear terms, but are nearly impossible to predict;

2) Chaotic systems are sensitive to initial conditions;

3) Complex Systems involve multiple component parts that interact in a nonlinear fashion; and

4) The results of Complex Systems are often greater than the sum of their parts.

Examples of chaotic systems include the weather, war, love, population growth, many epidemics and stock market prices. Chaos Theory has been used to explain psychologic health as well as specific health behaviors such as smoking and physical activity [15-17].

One of the first published works on Chaos Theory came from a meteorologist named Edward Lorenz. In the 1960's he was developing computer models of weather prediction. One day after running a predictive equation he decided to run the model a second time. But to save time he started the calculation in the middle of the sequence, plugging in manually some key numbers. But the predicted output diverged sharply from the original. He eventually discerned that in the original computation the number used was .506127 but in the simulation he had only entered the first three digits, .506 [18]. This phenomenon, eventually labeled "sensitivity to initial conditions", posits that a minor change at the beginning (or at various points) of a sequence of events can dramatically alter the long-term outcome of the system. This is commonly referred to as the butterfly effect.

The flapping of a single butterfly's wing today produces a tiny change in the state of the atmosphere. Over a period of time, what the atmosphere actually does diverges from what it would have done. So, in a month's time, a tornado that would have devastated the Indonesian coast doesn't happen. Or maybe one that wasn't going to happen, does. (Ian Stewart, Does God Play Dice? The Mathematics of Chaos, pg. 141) [19].

The weather is considered a classic chaotic system, as described in the text below. Yet, simple substitution of health behavior terminology for meteorological terminology reveals striking similarity.

The weather (**BEHAVIOR CHANGE**) is an example of a chaotic system. In order to make long- term weather forecasts (**PREDICTIONS OF BEHAVIOR CHANGE**) it would be necessary to take an infinite number of measurements, which would be impossible to do. Also, because the atmosphere (**HUMAN BEHAVIOR**) is chaotic, tiny uncertainties would eventually overwhelm any calculations and defeat the accuracy of the forecast. Even if it were possible to fill the entire atmosphere of the earth with an enormous array of measuring instruments, e.g., thermometers, wind gauges, and barometers (**PSYCHOSOCIAL, BIOLOGIC, AND ENVIRONMENTAL MEASURES**) uncertainty in the initial conditions would arise from the minute variations in measured values between each set of instruments in the array. Because the atmosphere (**HUMAN BEHAVIOR**) is chaotic, these uncertainties, no matter how small, would eventually overwhelm any calculations and defeat the accuracy of the forecast (**PREDICTION**).

Another metaphor for sensitivity to initial conditions involves rolling two identical balls down a tall rocky mountain. Starting the balls even an inch or less apart at the top of the mountain could result in the two balls ending hundreds of feet apart at the bottom; having traversed vastly different courses. The different pathways created by slight differences in the impact point on a billiard ball is another example.

One additional concept from Chaos Theory, fractal patterns, may also be relevant to understanding human behavior. Fractals, which have been identified in natural science in the mapping of the microvascular system and snow flake geometry, are recurring patterns within larger systems that are self-similar, that is, a shape appears similar at all scales of magnification. In terms of human behavior, there may be common patterns of behavior change within and across individuals that follow certain complex, non linear patterns. Thus, although behavior change may unfold in an almost infinite combination of knowledge, attitude, efficacy, and intention, there may be recurrent patterns of change that may be used to identify audience segments which could be targeted by common interventions.

### Application to health behavior

Health behavior may mirror other Complex Systems found in nature in that they involve multiple component parts that interact in a nonlinear fashion. Factors such as knowledge, attitude, belief, and efficacy no doubt exert some influence on health behavior change. However, the interaction of these factors represent a complex system bound by chaotic regulation. For example, which particular bits of knowledge, attitude, belief, etc and the amount of each required to "tip" the system for a particular individual is virtually impossible to predict, and the outcome is sensitive to initial conditions. Initial conditions within individuals, e.g., relevant prior experience with a particular disease (e.g., family history) or a genetic predisposition may alter the interaction in profound ways. And, the slightest change in the system, i.e., the addition of one more piece of information or persuasion could dramatically alter the outcome. Such complex relationships are well represented by the swirling patterns created by mixing multiple colors of dye with a stick.

Given the non-linear nature of complex systems they are usually represented mathematically by quadratic or other non-linear models. In the linear framework unaccounted for variance is generally relegated to the catch all "error" term, when in fact such "error" may represent the chaotic component of the outcome. Stated otherwise, "error" may be the result of imposing a linear model on a non-linear phenomenon. Additionally, in complex dynamic systems the interaction of factors can yield almost infinite potential patterns. In linear terms, this may be analogous to higher order interaction terms that could involve 5, 10, or 15-way interactions. Although linear methods can be used to model such interactions, they are limited statistically and conceptually. First, the ability to detect such interactions would be underpowered, so unless the magnitudes of these interactions are pre-specified so that the study could be adequately powered, these analyses would generally lead one to assume, perhaps falsely, that no interaction exists. Second, untangling a 3-way or higher order interaction generally extends beyond our ability to map and interpret such a finding; a relatively simple two-way interaction states that the effect of one variable on the outcome is not constant, but depends upon the level or status of yet a second variable (e.g. the intervention effect on cholesterol reduction is not constant, but is greater for males that for females). The extension to a 3-way interaction says that that observed gender by treatment interaction is itself not always better for males than for females, but depends upon the status of a third variable (perhaps the intervention does better for tall males, but no better than it works for short females, etc). And this is a reduced example where each variable in the interaction only has two levels. In complex systems the levels of interactions are copious. Finally, from a chaotic perspective the confluence of interactions both within and between individuals is highly variable and the system is sensitive to initial conditions making prediction of such complex interactions virtually impossible. From a chaotic perspective, rather than searching for main effects or simple 2-way interaction effects, behavior change is assumed to involve multiple levels of interaction that vary across individuals. Linear models of behavior change are then both conceptually inappropriate and statistically futile. In traditional statistical terms this would equate to analyzing and reporting separate main effects for multiple independent variables when there are known interactions (non linear in nature) of these variables. The solution does not do justice to the complexity of the phenomena.

A potential important element of this model that should also be considered is the occurrence of random external and intrapsychic events. Chaotic systems are not synonymous with randomness, nonetheless, random events can significantly impact complex systems. Consider why after years of false starts and failed attempts, a person succeeds at increasing their physical activity, eating healthier or losing weight. Or, why after years of success a person relapses. One explanation is that success or failure is determined by random events. The event may be external, such as hearing about someone they knew who lost weight, quit smoking, or perhaps passed away. This is similar to the "Cues" concept in the Health Belief Model [20,21]. The random event may also be intrapsychic. Without conscious thought, the person may experience a surge of motivation that they need to and/or are able to change or a craving may arise unexpectedly that triggers a relapse. Such feelings may be stimulated by associations created by classical conditioning about which the individual may not be conscious. Regardless, motivation and impulse arrives as opposed to being planned.

Consistent with this perspective, West et al recently reported an analysis of how smokers decided to quit. Approximately half of the ex- and current smokers in their sample reported that their most recent quit attempt was unplanned and those who did quit this way were more likely to stay quit than those who made a specific plan to quit [22]. Another study of smokers found that more than half of quit attempts were spontaneous rather than planned [23]. West et al explain their findings using "catastrophe theory" [22], which posits that dramatic outcomes can result from continuous pressure of a force on a system. An example often used to illustrate this concept is the result of gradually bending a plastic ruler until it snaps or the point at which water becomes vapor. So too, motivation may break or boil when enough pressure is applied to the system.

Chaotic patterns can stimulate behavior change in two distinct ways. In the first, single external random events such as a conversation, a public service announcement, newspaper article, word about the death of a friend or relative, etc may serve as a tipping point for motivational change. Conversely, absent an external event, resident chunks of knowledge or attitude may randomly coalesce to form a perfect motivational storm. Miller also delineates two types of quantum change, with one being more a dramatic, mystical experience and the second being more a sudden insight or sense of finding one's truth. Common to both pathways is that they occur outside of conscious reasoning; that they happen to the person [13]. As Miller notes, the individual experiences a "fast forward to self actualization". Interestingly in a study of problem drinkers, those whose decision to quit drinking arose from a transformational experience (having experienced a negative/traumatic event such as hitting rock bottom or having a spiritual awakening) were twice as likely to be non-problem drinkers at followup whereas those who reported weighing the pros and cons of drinking were actually more likely to have drinking problems at followup [24]. The cognitive approach to behavior change in this study was associated with worse outcomes. Thus not only do there appear to be linear and quantum pathways to change, the two processes may impact behavioral outcomes differently.

Another perspective that may be useful to include in this alternative paradigm is the concept of "Tipping Points". Tipping points are dramatic changes in social behavior that arise quickly and usually unexpectedly [25]. Whether it be a jingle or slogan; a political idea or mass purchase of a "fad" product, such tipping points are virtually impossible to predict, yet retrospectively coherent explanations for the phenomena are routinely offered. Similarly, each night after the stock market closes, pundants explain why certain events of the day or week "caused" the price fluctuations. Yet, a priori, few pundants could have predicted the impact of said events. If they possessed such prognostication ability they would be extremely wealthy. The stock market provides an excellent metaphor for chaos, as on an almost daily level, tipping points occur that lead to what has been called the random walk theory of wall street [26]. Additionally, just as our interventions often work, the stock market tends to rise. The former may be due to an inherent will to live and the latter inherent optimism of consumers. However, in both cases, there may be underlying human dynamics that predispose systems to moving in a particular direction.

Threshold effects or tipping points are commonly used in epidemiology. For example cutpoints for obesity, hyperlipidemia, and blood pressure are in part based on non-linear thresholds at which disease risk begins to rise at a faster rate [27]. In behavioral terms, the tipping point refers to the threshold at which individuals or groups of individuals adopt a particular idea or practice. Relating this to the obesity epidemic for example, there may be a societal tipping point at which a large percent of the population decides to alter their diet and activity patterns. A recent tipping point occurred in 2004–2005 when as much as 15% of the US population had tried the Atkins diet or some other low carbohydrate regimen [28], despite little scientific evidence demonstrating effectiveness [29-31]. Such non linear shifts have also occurred in the prevalence of smoking and illicit drug use [32,33]. However, they are difficult to predict let alone cause.

It is important to note that the chaotic perspective of behavior change offered here focuses mostly on the individual intrapsychic dimension. Environmental factors such cost, availability, legal restrictions etc also interact with intrapsychic determinants. In some cases, environmental determinants can overwhelm system constraints. For example, raising cigarette taxes by several dollars per pack, has a suppressing impact on individual smoking behaviors, whereas lack of availability of fruits and vegetables can constrain dietary choices.

#### Resistance to Chaos

Accepting randomness as a primal determinant of human behavior may be contrary to the deterministic view characteristic of western thought. Randomness may conflict with an innate tendency for humans to infer causality and a need for predictability. For example, when a punter wins the lottery, a completely random event, many individuals will assume that the winner used some replicable strategy that led to them to "earn" their prize or that some higher order "kharma" deemed the winner worthy. Accepting randomness requires that we relinquish the faith that reward and punishment; fortune and misfortune are doled out in an orderly, just fashion. Perhaps not surprisingly, Chaos theory and non-linear dynamics have met considerable resistance within the scientific community [18]. For public health professionals it requires a new conceptualization of health behavior as well as how and why we influence change.

In the complex system approach, the role of health communications may be analogous to the spinning of ping pong balls in a lottery machine. Say that each ping pong ball represents a chunk of knowledge, attitude, efficacy, or intention. On each ball lies a few strips of Velcro; the soft side. Inside the human psyche lies strips of the opposite, hard side of Velcro, which serve as potential motivational "receptors". Some of the motivational ping pong balls may have resided in the system for years while others may have been more recently implanted through a health education program, clinical counseling encounter, or health communication campaign. Rather than attempting to predict which piece or pieces of motivation may "tip" the individual, from the chaotic perspective, the role of the health professional is to ensure the balls are kept spinning at various intervals and velocities to maximize the chances that they adhere to their receptors. When sufficient balls have adhered a tipping point may occur. Which balls or combination of balls may trip the motivational switch as well as when and why they may stick, are chaotic events that defy accurate prediction. From a non-linear perspective, the goal of health professionals may be to encourage wing flapping.

The linear and chaotic paradigms are not necessarily mutually exclusive. Behavior change includes both chaotic and rational processes. As shown in the figure below, the Cognitive-Planned and Chaotic-Quantum aspects of motivation can be placed along a continuum. The continuum may be seen as a framework to both classify motivational styles (across individuals) or behavioral decisions (within individuals).

Some individuals may by their nature be prone to employ rationale decision making processes typically associated with left hemispheric function. On the other hand some may be more predisposed to quantum processes where change is more dramatic and less planned. Most individuals are likely influenced by both linear and quantum processes, perhaps depending on mood or other initial conditions. Another way to conceptualize the interaction of linear and quantum processes is that cognitive-rational factors may provide the fertile soil on which chaotic events may sprout. Thus, health promotion may be viewed as priming individuals so that when chaotic environmental or intrapsychic events occur, they have a greater likelihood of taking root. Whether individuals possess a predisposition to either style is an important issue with considerable implications for health communications. If valid, one implication is that program planners may need to tailor intervention content and delivery to match individual cognitive/motivational styles. Whereas quantum processes may be more operative at initiation of change, it is possible that cognitive-rational processes may be more relevant to maintenance of behavior change.

### Summary and Implications for Practice and Future Research

The random component of health behavior change, though difficult to predict or control, can nonetheless be incorporated into practice and research. For example, using the "perfect storm" analogy, it may be important to provide individuals with periodic interventions so that the motivational ping pong balls are spun under varying "atmospheric" (i.e., psychologic and/or life circumstances) conditions. Periodic exposure is consistent with the approach used in many chronic disease management programs. Such program, from this new perspective can be viewed as providing repeated opportunities to produce the motivational storm. This approach is also consistent with counseling models such as motivational interviewing, which provide clients with considerable opportunity to explore life with and without their risk behavior; that is to spin the balls [34,35].

Another implication is that individually tailored interventions may be particularly promising as a means to maximize the likelihood of a perfect motivational storm [36-40]. Individually tailored communications increase both receiver attention and message salience, which together increase the chances that the "balls" are spun and that they have a optimal chance of sticking.

There are also statistical implications. The potential variance in behavior accounted for by traditional cognitive factors should perhaps be assumed to have an upper limit far below 100%. Given prior studies, a reasonable upper limit may be in the 50% range. And rather than assuming unaccounted for variance simply reflects "error", non-linear models could be used to explore alternative mathematical relationships. And although the relationship of predictor variables may be complex and non-linear, there may be identifiable patterns, i.e., fractals in the parlance of chaos theory, that manifest across individuals that would allow for sophisticated audience segmentation and potentially powerfully tailored interventions.

We are not proposing that linear statistical models and linear-based health promotion interventions are of no value and need be discarded entirely. There is a vast scientific base indicating that our interventions can successfully change behavior. What we are proposing, however is that we need to rethink why our interventions work and for whom. Group interventions, we propose, work because they have spun the "balls" of motivation (or deactivated barrier balls) in a large group of individuals, and for a subset of these individuals the balls fit their motivational receptors and other psychologic and biologic settings. It is important to note that current theories and communication methodologies can greatly inform which "balls" we select to highlight in our interventions. Motivation is not random. Tailoring motivational messages to the audience remains a critical step in achieving positive outcomes, and our current theories can help select the most effective set of balls.

Additionally, although patterns of change likely follow unique, i.e., chaotic, patterns across individuals, it may nonetheless may be useful to know that, in aggregate, balls that have similar characteristic profiles tend to "pool" in a defined geographic area once rolled down the metaphorical intervention mountain, helping us to perhaps understand which ping pong balls to keep circulating and for whom. That is, there may be common pathways to change based on individual parameters that can be used to develop sophisticated audience segmentation analyses and more effective interventions that account for the chaotic element of change. A "mixture model" of both chaotic and linear progression may be one that helps us best understand change.

The proposition that a significant proportion of human behavior operates from a chaotic perspective, at first blush, may appear to defy empirical verification. However, with the advent of technologies such as Functional Magnetic Resonance Imaging (fMRI) and momentary psychologic assessment, it may be possible to examine where, neurologically different types of motivation arise, and even predict when and why quantum transformations occur. Theoretical and statistical research examining behavior change from a quantum perspective is encouraged. In particular, the degree to which transformational motivation observed in the addiction field operates in the nutrition and physical activity domains, and whether changes spurred by inspiration are more enduring than changes arrived at from the more cognitive, conscious pathway merits examination.

**Figure 1:**
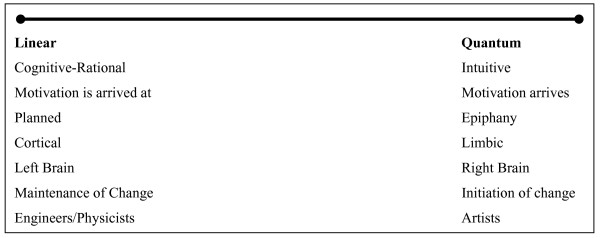
Continuum of Motivational Processes.
